# Conductive Polyaniline Patterns on Electrospun Polycaprolactone/Hydroxyapatite Scaffolds for Bone Tissue Engineering

**DOI:** 10.3390/ma14174837

**Published:** 2021-08-26

**Authors:** Izabella Rajzer, Monika Rom, Elżbieta Menaszek, Janusz Fabia, Ryszard Kwiatkowski

**Affiliations:** 1 Department of Mechanical Engineering Fundamentals, Faculty of Mechanical Engineering and Computer Science, University of Bielsko-Biala, 43-309 Bielsko-Biala, Poland; 2 Faculty of Materials, Civil and Environmental Engineering, University of Bielsko-Biala, 43-309 Bielsko-Biala, Poland; mrom@ath.bielsko.pl (M.R.); jfabia@ath.bielsko.pl (J.F.); rkwiatkowski@ath.bielsko.pl (R.K.); 3 Department of Cytobiology, Collegium Medicum, Jagiellonian University, 31-007 Krakow, Poland; elzbieta.menaszek@uj.edu.pl

**Keywords:** scaffolds, electrospinning, inkjet printing, conductive polymers, polycaprolactone, polyaniline

## Abstract

Currently, the challenge for bone tissue engineering is to design a scaffold that would mimic the structure and biological functions of the extracellular matrix and would be able to direct the appropriate response of cells through electrochemical signals, thus stimulate faster bone formation. The purpose of the presented research was to perform and evaluate PCL/n-HAp scaffolds locally modified with a conductive polymer-polyaniline. The material was obtained using electrospinning, and a simple ink-jet printing method was applied to receive the conductive polyaniline patterns on the surface of the electrospun materials. The samples of scaffolds were analyzed by scanning electron microscopy (SEM), X-ray diffraction (XRD), thermal analysis (DSC, TGA), and infrared spectroscopy (FTIR) before and after immersion of the material in Simulated Body Fluid. The effect of PANI patterns on changes in the SBF mineralization process and cell morphology was evaluated in order to prove that the presented material enables the growth and proliferation of bone cells.

## 1. Introduction

A variety of fabrication technologies have been implemented to produce an ideal biomaterial to treat bone fractures [1,2]. Fiber-based structures, such as electrospun scaffolds, are frequently used in bone regeneration as they mimic the architecture and biological functions of the extracellular matrix [3,4]. Ideally a scaffold should possess the following characteristics: (1) 3D and highly porous microstructure with an interconnected pore network; (2) be biodegradable or bioresorbable with a controllable degradation and resorption; (3) suitable surface chemistry; (4) mechanical properties that match those of tissue at the site of implantation; and (5) be quickly processed to form a variety of shapes and sizes [5]. The limited control of the pore size and pore structure are meaningful disadvantages of the electrospinning technique [6]. Tiny pores defined by densely compacted fibers considerably hinder cell infiltration and tissue ingrowth. Furthermore, the pore size of electrospun scaffolds is dependent on the fiber diameter. Fibers with a smaller diameter lead to smaller average pore sizes, driving to decreased cellular infiltration. This occurrence limits the potential benefits of nanofibers for bone tissue engineering applications [6]. However, to improve the porosity of the final scaffold, scientists have developed a different techniques combining electrospinning with other methods [7]. For example, to achieve control over electrospun fiber deposition, electrospinning in a direct writing (DW) mode was developed. This technique provides the combined benefits of the topography provided by electrospun mats with the reproducibility and designing potential of Additive manufacturing techniques. Electrospun nanofibers can also be used as a platforms for the development of more innovative in vitro models to study bone cell behavior, their proliferation and differentiation under different stimuli [6].

Electrical stimulation is a method commonly used in orthopedics to treat bone fractures [8]. The positive impact of electrical stimulation on bone tissue healing and regeneration has been demonstrated both experimentally and in clinical applications. Electrical stimulation can modulate and accelerate the action of nerves and skeletal muscles. Many in vitro and in vivo studies confirm the usefulness of electrical stimulation in bone healing at a cellular level. Researchers have shown that low voltage electrical stimulation stimulates bone cells to migrate, proliferate, align, differentiate, and adhere to biocompatible scaffolds [8]. As a result of this discovery, a novel generation of smart biomaterials, i.e., electroactive materials, is currently gaining prominence in tissue engineering [9].

Conductive polymers are materials that allow for the direct transference of electrical, electrochemical and electromechanical stimuli to cells. Researchers have studied a variety of conductive polymers such as: polypyrrole (PPy), polyaniline (PANI), polythiophene (PTh), and poly(3,4-ethylenedioxythiophene) (PEDOT) [10,11]. Conductive polymers offer advantages over conventional polymers due to ease of synthesis, and they also exhibit electrical and optical properties similar to those of metals and semiconductors [12]. Polyaniline is one of the most widely investigated conducting polymers thanks to its high stability, diversity of structural forms, low cost and controllable conductivity [13]. Meanwhile, PANI derivatives can be used in biomedical applications, most notably, drug delivery and tissue engineering [14]. However, PANI is challenging to process because of its poor solubility in most of the common solvents [15]. These limitations can be overcome by coating polymeric substrates with conducting polymers to make composites [16]. It is also possible to process conductive polymers into nano- and microfibers using the electrospinning technique. Via this process, conductive polymers can be electrospun alone or combined with spinnable polymers [17]. For example, polypyrrole has been compounded with poly(d,l-lactide) (PDLLA), while PANI has been blended with natural polymers such as gelatin and collagen and [18]. Polycaprolactone and its derivatives are one of the most studied synthetic biomedical polymers and according to the literature, a PCL/PANI blended solution has also been prepared to produce scaffolds suitable for skeletal tissue engineering, enhancing myoblast attachment, proliferation, and myogenic differentiation [19,20]. Electrospun scaffolds have been composed of electrically conductive nanofibers with highly oriented structures [21]. Other researchers have proved that a bioactive and patterned nanofiber scaffold provides both topographical and biochemical cues to induce, enhance, and guide neurite outgrowth [22].

The aim of our study was to obtain composite conductive biomaterial for scaffold purposes that could serve as a platform for bone tissue regeneration stimulated by electrical impulses. In this study, hydroxyapatite particles were incorporated into polycaprolactone fibers in order to regulate osteoinductivity of the scaffolds and stimulate bone formation. Thin patterns of polyaniline in the form of ink were deposited on electrospun materials using the inkjet printing system. By coupling conductive PANI with PCL/n-HAp electrospun fibers, we wanted to achieve a bioactive, electrically conductive and biomimetic environment for the cells. We evaluated the effect of the PANI patterns presence on scaffold properties as well as the mineralization process in Simulated Body Fluid. We also investigated the morphology and osteogenic differentiation of NHOst cells cultured on produced scaffolds. Conductive patterns obtained in this study will allow for an external electrical stimulation that could be applied to the cells, thereby controlling the growth, proliferation, migration and differentiation of the cells.

## 2. Materials and Methods

### 2.1. Electrospinning

A polycaprolactone (PCL) (Mw 80 kDa, Sigma-Aldrich, Poznań, Poland) solution was prepared by dissolving 2.5 g of PCL into a 40 mL of chloroform/methanol (1:1) mixture (POCH, Poland). To the PCL solution, 0.5 g of carbonate nano-hydroxyapatite (n-HAp, AGH, Kraków, Poland) powder was added [23]. A stable dispersion of n-HAp was achieved through the sonication of the solution. The prepared solution was poured into a plastic syringe (10 mL) with 18-gauge blunt tip needle and was subjected to the electrospinning process. The nanofibers were electrospun using a TIC 1092012 device (Bielsko-Biala, Poland). Numerous tests were carried out to in order to determine the parameters of the electrospinning process and to obtain fibers with suitable morphology. Finally, the following process parameters were selected: a high voltage of 30 kV, the flow rate of 1.5 mL/h, and a distance from the tip of needle to the collector of 15 cm. The nanofibers were then deposited onto the cylindrical rotating drum (230 rpm) which was wrapped in silicone-coated paper.

### 2.2. PANI Printing

**Figure 1:**
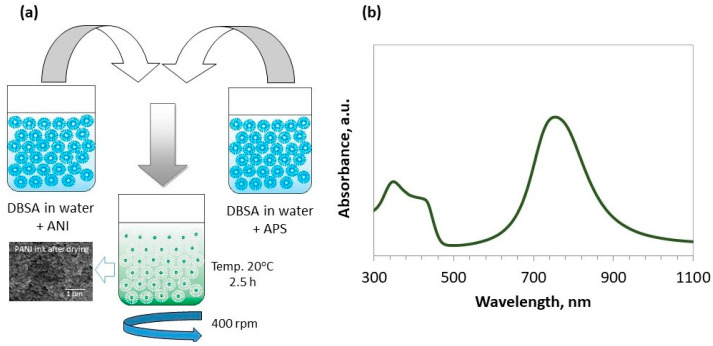
Synthesis of polyaniline. PANI is prepared in an aqueous medium with surfactant and using ammonium persulfate as an oxidizing agent (**a**). The UV-Vis spectrum of PANI ink in water (**b**).

Polyaniline ink was synthesized through the oxidative polymerization of aniline (Avantor Performance Materials, Gliwice, Poland) in water with ammonia persulfate (NH_4_)_2_S_2_O_8_ (APS) as the initiator of the reaction. The application of APS enabled us to obtain the greatest efficiency, conductivity and molecular mass of the PANI. The synthesis proceeded according to the scheme presented in Figure 1.

In order to obtain a stable dispersion of the PANI in the water-based ink, despite the DBSA, (PCC, Rokita, Poland) 0.1 M of sodium dodecyl sulfate (SDS, Avantor Performance Materials, Poland) was added. The ratio of ANI:APS:DBSA was 1:1:1. As-synthesized dispersion of the PANI in water was then subjected to dialysis for 24 h. UV-Vis spectroscopy (Perkin Elmer Lambda 35) was used to obtain information on PANI structure and conductivity. The spectrum presented in Figure 1b provides information on the presence of the conductive form of polyemeraldine with the typical maximum at 340 nm (π–π* transition) and polaron bands at ~765 nm and ~420 nm. Those polaron bands indicate a “coil-like” structure of the polymer chain. Conductive polyaniline patterns (ink: aqueous dispersion of polyaniline) were printed onto the nanofibrous substrates prepared by electrospinning using a Dimatix DMP 2831 material printer. The influence of the ink on the properties of the obtained materials was evaluated.

### 2.3. Scaffold Characterization

A Scanning Electron Microscopy (NOVA NANO SEM 200 (FEI Europe Company, Hillsboro, OR, USA), equipped with an energy dispersive X-ray (EDX) detector (EDAX), was used to characterize the microstructure of the electrospun samples before and after the printing of conductive patterns. The samples were coated with a carbon layer before observation. The diameter of the fibers were measured based on SEM images using Image J software. The average diameter of the fibers was determined by performing measurements on 60 fibers.

The attenuated total reflectance Fourier transform infrared (ATR-FTIR) spectra were measured using a FTS Digilab 60, BioRad spectrophotometer (in the range 500–3500 cm^−^^1^, with the resolution of 4 cm^−1^).

Differential Scanning Calorimetry (DSC) and thermogravimetry (TG) tests were carried out using the NETZSCH STA 449 F3 thermal analyzer, SDT 2960 TA INSTRUMENTS thermogravimetric analyzer and a DSC 2010 TA INSTRUMENTS differential scanning calorimeter. The tests were carried out at a heating rate of 10 °C/min under an inert gas (nitrogen) atmosphere with a gas flow of 40 mL/min.

WAXD analysis were carried out using a URD63 diffractometer (Seifert, Germany) equipped with a secondary beam graphite monochromator. The X-ray scattering measurements were carried out using the step method, in the 2θ scattering angle range from 2.5° to 60° in 0.1° steps, using an X-ray tube with a copper anode (CuKα radiation, λ = 1.542 Å) operating at a voltage of U = 40 kV, and current I = 30 mA.

The in vitro bioactivity evaluation of the obtained scaffold was carried out in a simulated body fluid (SBF) medium. The 1.5× concentrated SBF was selected to examine the mineralization process of PCL/n-HAp and PCL/n-HAp/PANI scaffolds [24]. Samples of the nonwovens (2.0 × 2.0 cm) were placed in 15 mL Falcon polypropylene containers and kept in 1.5× SBF for 2 weeks at 37 °C. The fluid was changed every 2.5 days. After 1, 3, 7 and 14 days of incubation, the samples were rinsed with distilled water and then dried at room temperature. The assessment of changes occurring in the materials was carried out using the SEM, FTIR and WAXD methods.

The percentage changes in the mass of the samples were calculated according to the formula:

Δm = (mp − mk)/mk·100%(1)

where: mp—mass of the sample before incubation; mk—mass of the sample after incubation.

Changes in the stability of samples immersed in physiological fluids were monitored by measuring pH (Elmetron Cp-401 pH-meter) and conductivity (Elmetron CC-505 conductometer). Ca^2+^, K^+^ and Na^+^ ion concentration in the SBF after various periods of sample incubation were determined using an ion analyzer (Medica EasyLyte Ca/K/Na/pH).

The effect of passing an electrical current through PCL/n-HAP/PANI scaffolds was investigated. Determination of the electrical properties of materials printed in the same way was carried out before based on the basing on the electrochemical impedance spectroscopy measurements (EIS) [25]. This time, in order to check the electrical conductivity, the effect of passing an electrical current through a PCL/n-HAp/PANI scaffold was investigated by means of the four- point probe method [26]. Samples were prepared by depositing PANI ink on the surface and then tested after drying. The distance between electrodes was 0.1 cm.

Normal human osteoblast cells (NHOst) (Lonza, Tampa, FL, USA) were used and cultured on obtained scaffolds. All tests were performed as described elsewhere [27]. Cell mineralization (OsteoImage test, Lonza, Tampa, FL, USA) and ALP activity (4-MUP) were evaluated on days 7, 14 and 21. Cell morphology was evaluated using an optical fluorescence microscope (Olympus, Japan) and a scanning electron microscope (NOVA NANO SEM 200, FEI EUROPE COMPANY). The cells were stained for 0.5 min with fluorochrome-acridine orange (AO), and rinsed with phosphate-buffered saline (PBS). For SEM imaging, cells were fixed for 30 min with a 3% solution of glutaraldehyde in a cacodylate solution at pH 7.4, washed with PBS, dehydrated by passing through an increasing series of alcohols, dried and sprayed with a thin layer of carbon.

#### Statistical Analysis

All of the quantitative results were expressed as mean ± standard error of the mean. Statistical analysis was carried out employing a one-way analysis of variance (ANOVA). A *p*-value less than 0.05 was recognized as statistically significant.

## 3. Results

**Figure 2:**
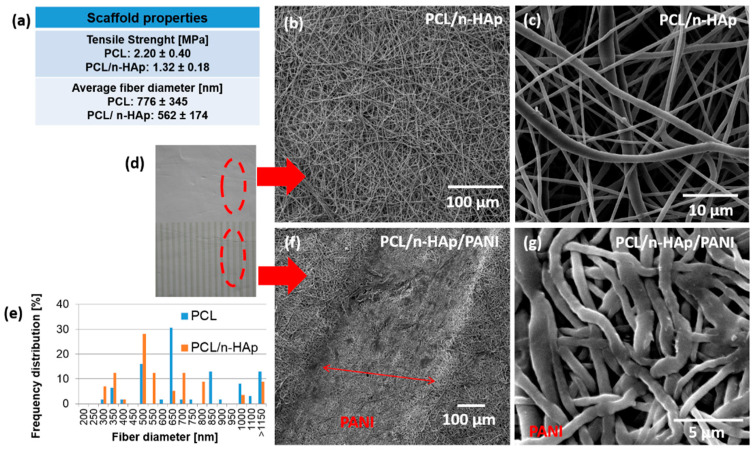
(**a**) Properties of the PCL/n-HAp scaffold. SEM images of the electrospun scaffolds: (**b**,**c**) PCL/n-HAp; (**d**) macroscopic image of PANI deposited on electrospun scaffold; (**e**) diameter of fiber distribution for PCL/n-HAp scaffold; (**f**,**g**) the layer of PANI deposited by inkjet printing on the surface of scaffold (PCL/n-HAp/PANI).

The electrospinning process was used to fabricate composite fibrous PCL/n-HAp scaffolds for further application in bone tissue engineering. The comparison of pure PCL and PCL/n-HAp scaffolds was performed in our previous study [27]. The data regarding the tensile strength of the modified and unmodified scaffolds, as well as average fiber diameter, are displayed in Figure 2a.

The diameter of PCL/n-HAp fibers were calculated (Figure 2e) based on the scanning electron microscope observation. The average fiber diameter was around 562 nm for PCL/n-HAp fibrous scaffolds and 776 nm for unmodified material. The tensile strength decrease ranges from 2.2 MPa for an unmodified nonwoven to 1.3 MPa for the composite PCL/n-HAp scaffold. To form conductive paths on the nonwoven materials, inkjet printing technology was applied. Figure 2d shows a macroscopic image of PANI patterns printed on the surface of electrospun nonwoven material. SEM images of the electrospun PCL/n-HAp (Figure 2b,c) and PCL/n- HAp/PANI (Figure 2f,g) fibrous membranes show that both membranes consisted of randomly oriented and bead-free fibers. It could be found that the morphology of fibers after inkjet printing was influenced by polyaniline as the fibers below the patterns became more flattened and the fabric became more dense (Figure 2g). Fibers in the material are very delicate and even a slight load on them causes deformation. The droplet of ink acts as the loading, so that the surface of the sample printed according to the designed pattern is deformed as it can be observed in Figure 2f.

**Figure 3:**
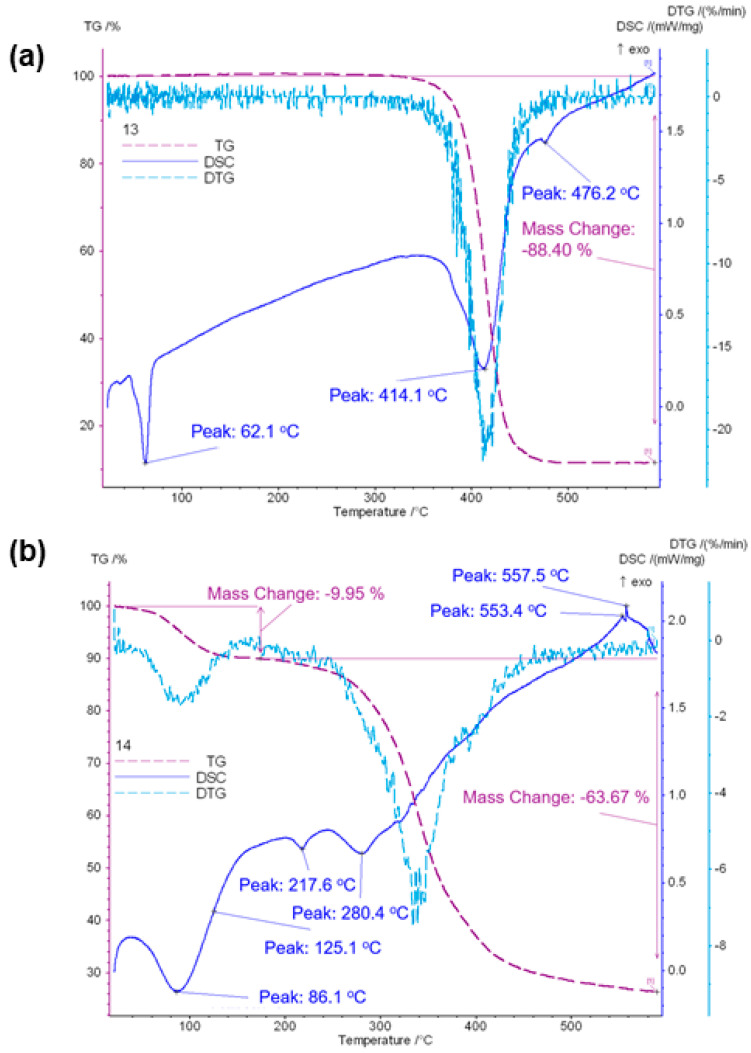
DSC, TG, DTG thermograms of PCL/n-HAp (**a**) and PCL/n-HAp/PANI (**b**) scaffolds.

The endothermic effect on the DSC curve of PCL/n-HAp nonwovens obtained by electrospinning, with the maximum occurring at 62 °C, is attributed to the melting of polycaprolactone (Figure 3a) [28].

The melting temperature Tm is the temperature at which the final disappearance of the crystalline phase occurs. The second largest maximum on the curves, occurring at 414 °C for PCL/n-HAp, is associated with a loss of mass (88.4%) and corresponds to polymer degradation. In the case of the PCL/n-HAp/PANI sample, we could observe the glass transition temperature of PANI around 86 °C (Figure 3b). Polymer degradation was also observed in the case of PANI modified samples. However, in the case of PCL/n-HAp samples modified with PANI patterns, a two-step degradation process (at 218 °C and at 280 °C) was observed. TG results showed the incorporation of PANI patterns into the electrospun scaffolds (Figure 3). It can be observed that the onset of scaffold degradation occurs at lower temperatures for PCL/n-HAp/PANI than for PCL/n-HAp scaffolds. This lower thermal stability is related to the presence of PANI and residues of DBSA. The first step of weight loss is between 40 and 140 °C for PCL/n-HAp/PANI scaffolds. This first step of weight loss is induced by moisture and DBSA acid, which is used as a solvent and dopant to prepare the PANI. The next step of weight loss in the range of 140–280 °C may be assigned to the oligomer degradation produced during the polymerization of PANI. The following weight loss step for the PCL/n-Hap/PANI sample is observed in the range 280–380 °C, which is caused by the degradation of Coulomb attraction between the sulphonic acid (DBSA dopant) and PANI chain backbone, due to the evaporation and degradation of the acid [29,30].

The bulk resistivity, and hence the conductivity of the scaffolds modified with PANI, was measured using the four point probe method and found to range from 0.2 to 0.3 mS cm^−1^, which was low but comparable with other results revealed by inkjet printing [31]. The high level of surfactant in the dispersions (necessary for the stabilization of the spherical nanoparticles during the synthesis and in the ink) as well as uneven thickness of printed layer might be the reason for this.

**Figure 4:**
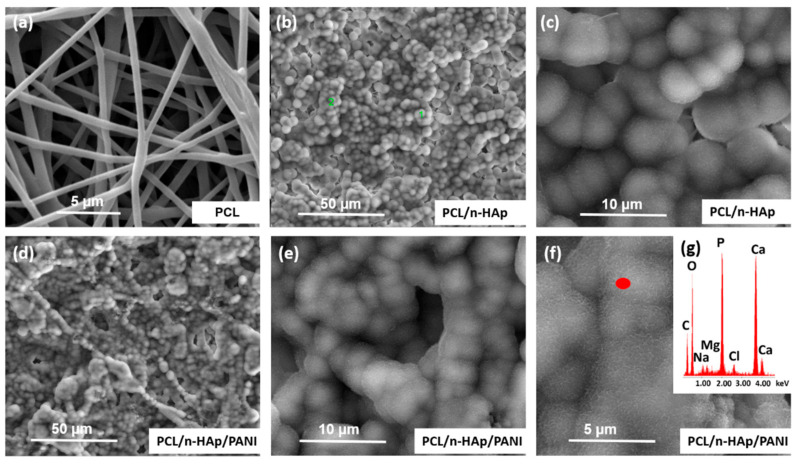
SEM images of: (**a**) PCL, (**b**,**c**) PCL/n-Hap, (**d**–**f**) PCL/n-Hap/PANI scaffolds after 14 days of immersion in SBF fluid together with (**g**) EDS analysis.

The mineralization behavior of the scaffolds was assessed by immersing them in 1.5× SBF solution for a period of up to 2 weeks. It can be observed that, before immersion, the sample shows a relatively flat and smooth surface morphology (Figure 2). During immersion treatment, the shape of the materials was maintained, and the materials did not dissolve. SEM images and EDX analysis of PCL/n-Hap and PCL/n-Hap/PANI composite samples after treatment in SBF solution are shown in Figure 4.

During incubation in artificial plasma, spherical apatite precipitations were observed on the surface of both samples. After 7 days of incubation in SBF, the early stages of crystallites of calcium phosphate and calcium carbonate were observed on both materials. In contrast to the modified samples, the apatite formation was not observed in the case of the unmodified PCL (Figure 4a), and the subsequent 7 days of immersion (after day 14), provided more calcium phosphate depositions observed on the surface of the scaffolds, confirming a process of deposit growth (Figure 4b–f). The development of a new, mineral phase on the scaffold surfaces was verified using EDX analysis, which confirmed the presence of calcium (Ca), and phosphorus (P) elements in the tested materials, thereby proving the presence of apatite on the surface of the PCL/n-HAp and PCL/n-HAp/PANI samples. The presence of PANI pathways did not disturb the arrangement of apatite on the surface of the samples.

**Figure 5:**
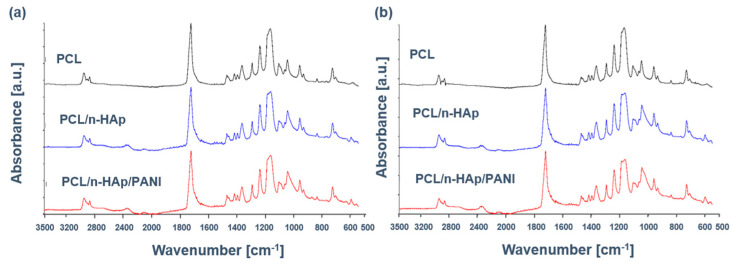
FTIR of scaffold: (**a**) before and (**b**) after immersion in SBF.

In this study, PCL/n-HAp and PCL/n-HAP/PANI samples before and after immersion in SBF were also analyzed using the FTIR technique, which is one of the primary methods for studying molecular structures and intermolecular interactions. FTIR spectra of pure PCL and PCL/n-HAp samples before and after application of polyaniline patterns on the nonwoven surface are presented in Figure 5a.

The spectrum of PCL presents a set of typical bands at 1157 cm^−1^ and 1293 cm^−1^ (C−O, C−C stretching), 1175 cm^−1^ and 1240 cm^−1^ (C−O−C, symmetric and asymmetric stretching, respectively) strong absorption band at 1727 cm^−1^ (C=O, stretching), 2865 cm^−1^ and 2949 cm^−1^ (CH_2_, asymmetric stretching). Although spectra of the PCL/n-HAp comprise the leading characteristic bands of PCL, some supplementary bands emerge, proving the presence of n-HAp. These new bands are located at 605 and 566 cm^−1^ (ν4 PO_4_^3−^) and 1033 cm^−1^ (ν3 PO_4_^3−^), although the PCL spectrum overlapped this band.

The characteristic bands of PANI are shown in Table 1.

The most important characteristic bands for PANI overlap with the polycaprolactone and hydroxyapatite bands. A small band at 3000–2840 cm^−1^ is assigned to a hydrophobic tail in DBSA. The small peak at 1559 cm^−1^ and a peak at 1477 cm^−1^ are attributed to C=C stretching vibration of quinoid and benzenoid rings, respectively. The peaks at 1146 cm^−1^ and 874 cm^−1^ can be assigned to the C-H in-plane bending vibration and the out-of-plane vibration of the 1,4-disubstituted benzene rings, respectively [15,32].

Figure 5b presents the ATR-FTIR spectra of scaffolds after immersion in SBF. After 14 days of immersion, PCL/n-HAp and PCL/n-HAp/PANI spectra exhibit characteristic phosphate bands, thus giving evidence of apatite formation. The absorption bands of phosphate groups were observed between 1100 and 1000 cm^−1^ and at 605 cm^−1^, 566 cm^−1^, corresponding to the different forms of PO_4_^3−^ groups in apatite. The broadening of the peak at 1033 cm^−1^ showed a polycaprolactone and its interaction with PO_4_^3−^ groups. The bands indicate the formation of a layer of an apatite on the surface of the samples during incubation in artificial plasma. It has been observed that the presence of conductive paths does not interfere with the formation of apatite on the surface of the produced scaffolds.

**Figure 6:**
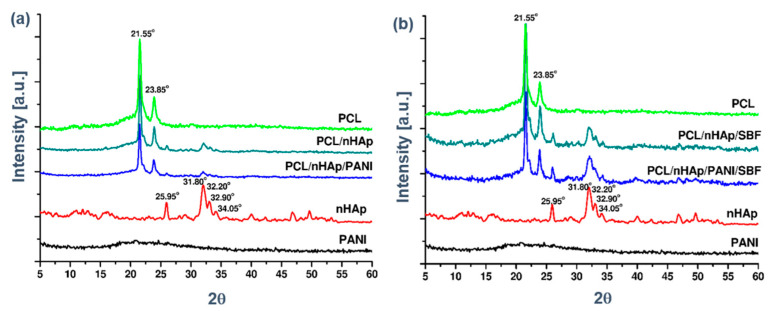
X-ray diffraction patterns for the PCL, PCL/n-HAp and PCL/n-HAp/PANI samples (**a**) before and (**b**) after 14 days of immersion in SBF.

The XRD patterns of the materials before and after soaking in SBF for 14 days are shown in Figure 6.

WAXD diffraction curves are characterized by the appearance of two strong diffraction peaks with maximums at 2θ 21.55° and 23.8°, typical for polycaprolactone, and with the presence of four peaks typical for hydroxyapatite at 2θ: 25.95°; 32.20°; 32.90°; 34.95° (Figure 6a). The presence of peaks, characteristic of each of the components of the PCL/n-HAp nonwoven fabric, indicates the presence of a two-phase structure in the fibers. After 14 days of immersion, the two peaks of apatite were intensified, indicating a formation of an apatite layer (Figure 6b).

**Figure 7:**
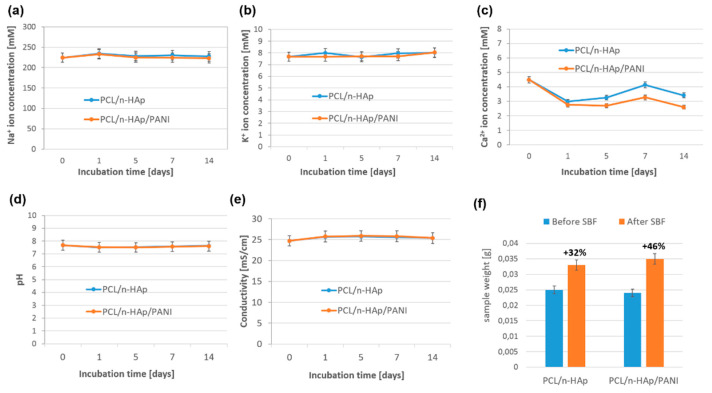
(**a**–**c**) The Na+, K+, Ca^2+^ ion concentration during incubation of the samples in SBF; (**d**,**e**) pH and conductivity of the SBF solution during incubation of samples. (**f**) The weight of the samples before and after incubation in the SBF fluid.

The studies on the chemical stability of samples immersed in the SBF solution have shown that, in the case of PCL/n-HAp, PCL/n-HAp/PANI samples, calcium ion concentration decreased after the first day of incubation (Figure 7c).

The decrease in the concentration of calcium ions was associated with the formation of the Ca-P layer on the surface of the nanofibers, and with the uptake of calcium ions from the solution. A slightly higher increase in calcium ions was observed in PCL/n-HAp than in PCL/n-HAp/PANI samples. The ion concentration of Ca reached a maximum point after 7 days, and then decreased slightly. The decrease and increase in ion concentrations in the SBF is as a consequence of the precipitation and dissolution processes when a bioactive composite material is immersed in SBF for 2 weeks. The concentration of sodium and potassium ions remained unchanged throughout the entire period of the incubation of samples in SBF (Figure 7a,b). There were no significant changes in the pH and conductivity of the SBF fluid during the two week incubation period (Figure 7d,e). The weight of the samples before and after incubation in the SBF fluid is shown in Figure 7f. The increase in the weight of PCL/n-HAp and PCL/n-HAp/PANI composite samples during the first 2 weeks of incubation was related to the formation of the apatite layer on the surface of the nanofibers. A higher increase in sample weight was observed in the case of the PANI modified sample. A major advantage of the printed patterns was their good adherence to the electrospun scaffolds when exposed to SBF aqueous media. The results of an in vitro study in SBF fluid have been confirmed using SEM examination (Figure 4).

**Figure 8:**
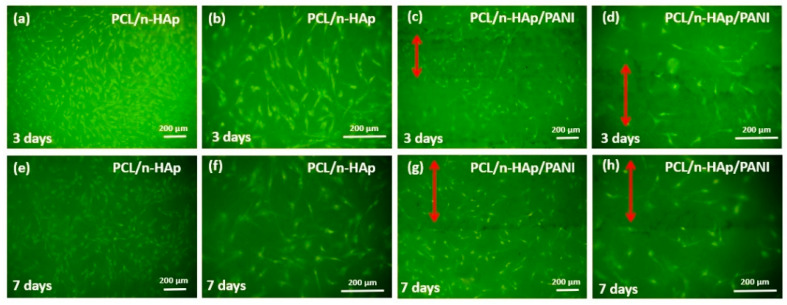
Morphology of NHOst cel culture for (**a**–**d**) 3 and (**e**,**f**) 7 days on the PCL/n-HAp electrospun materials (**a**,**b**,**e**,**f**) and PCL/n-HAp/PANI materials (**c**,**d**,**g**,**h**). Scale bar is 200 μm. Red arrows indicate areas with polyaniline applied.

The microscopic results of cells cultured on the PCL/n-HAp and PCL/n-HAp/PANI samples are presented in Figure 8.

The NHOst cells cultured on both types of samples had normal morphology and the cells were connected to each other via extensions, which allow for direct cell communication. Images showed a significant number of cells spread across the surface on both materials. The presence of PANI patterns does not negatively influence cell culture. Results of viability tests are presented in the supplementary file.

**Figure 9:**
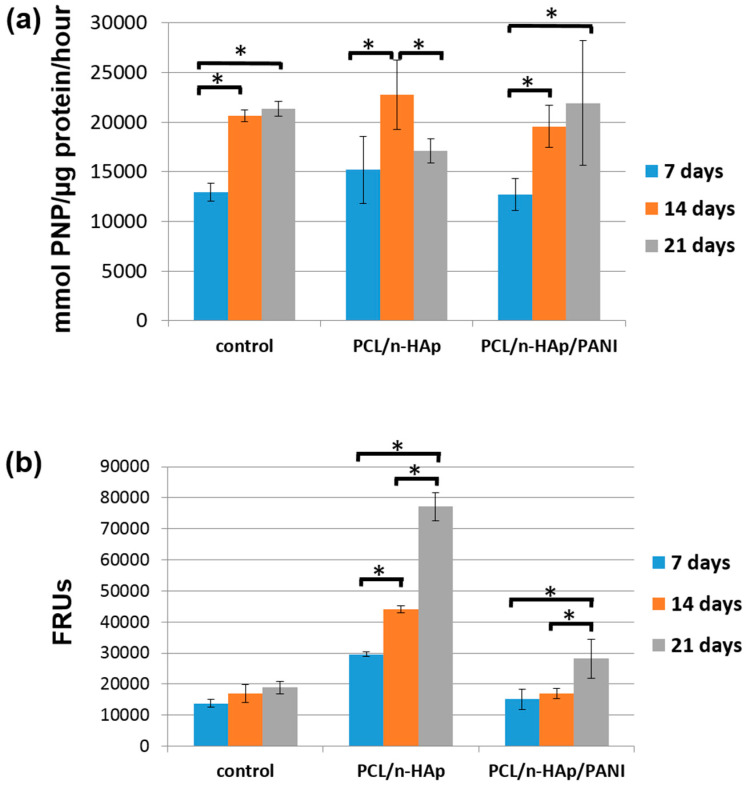
(**a**) Alkaline phosphatase activity test; (**b**) Mineralization progress measured by concentration of hydroxyapatites of NHOst cell culture on obtained materials. *: the results are statistically significant.

The differentiation level of NHOst cells was estimated by measuring alkaline phosphatase (ALP) activity and the amounts of calcium deposited. The early osteoblast differentiation of NHOst cells cultured for 7, 14 and 21 days under osteogenic media was examined through ALP staining. The results are presented in Figure 9a.

**Figure 10:**
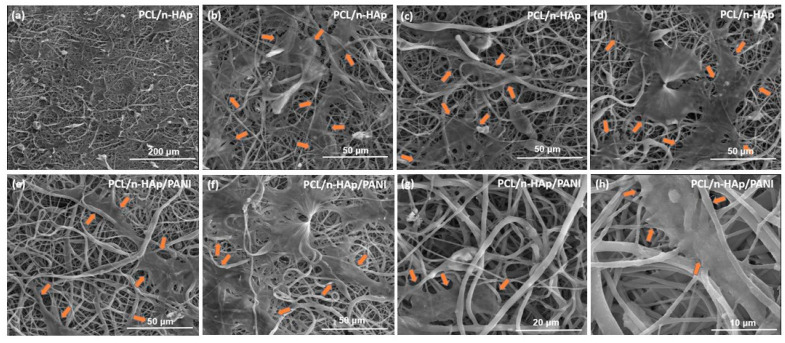
Scanning electron microscopy images of normal human osteoblast (NHOst) cells adhered to PCL/n-HAp (**a**–**d**) and PCL/n-HAp/PANI scaffold after 7 days of cell culture. The edges of growing cells are marked with arrows (**e**–**h**).

Alkaline phosphatase is a marker of early osteoblast differentiation, hence the high values of the tests determining its activity are still observed on the 14th day of culture, while after 21 days, ALP activity in cells growing on PCL/n-HAp/PANI and the control group increases only slightly. In the case of the PCL/n-HAp material, ALP activity after 21 days is significantly lower than in the case of the 14-day series with a high mineralization test result presented in Figure 10b (indicating faster cell differentiation). A statistically significant increase in ALP activity in the 14-day series compared to the 7-day series is evidence of the differential influence of the tested scaffolds on osteoblasts. Cell differentiation is also confirmed by OsteoImage (OI) test results presented in Figure 9b. The progress of mineralization was better pronounced in materials modified with nanohydroxyapatite without PANI patterns. However, after 21 days of cell culture on PANI patterns a high mineralization capability for these materials was also confirmed. A statistically significant increase in the OsteoImage test value, compared to the control for the PCL/n-HAp/PANI sample after 21 days of culture, indicates cell differentiation. Thus, the obtained results indicate the potential of the tested materials in initiating the cell differentiation and cell mineralization process.

The SEM images for the electrospun samples showed that the cells were spread out in an elongated morphology on the fibrous surface. Filopodia extensions are clearly visible in Figure 10.

This indicates that the surface was suitable for cell attachment.

## 4. Discussion

Our studies demonstrated that the combination of two methods for the preparation of scaffolds, i.e., electrospinning and inkjet printing, has enabled the production of nanofibrous, biomimetic scaffold with conductive paths on the surface. Previously, we succeeded in formation of conductive patterns on PCL/gelatin electrospun nonwovens composed of microfibers [25]. The fibers characterized within this study are almost half the thickness. With carbonate nano-hydroxyapatite as nanofiller, the electrospun PCL/n-HAp nanofibers were uniform and medium-sized in diameter. As the parameters of the electrospinning process affect morphologies of the fibers, even a little amount of nano-additive can influence the solution characteristics of PCL and disturb uniform formation of the fibers [33]. The tensile strength decrease in composite PCL/n-HAp scaffold is attributed to agglomeration of n-HAp, but one can also consider the weak interfacial adhesion between hydrophobic PCL and hydrophilic n-HAp [34]. Electrical stimulation is a method commonly used in orthopedics for treatment of bone fractures. The literature also describes the positive impact of the electrical potential on the regeneration of bone tissue. Creating conductive paths on the surface of the scaffold should allow the electrical stimulation of bone cells during tissue cultivation. The literature indicates that conductive polymers can modulate and accelerate the action of nerves, skeletal muscles, and even have a stimulating effect on bone cells. Thus, by modifying scaffolds using conductive polymers, it will be possible, using the electric current, to create the appropriate cell response (stimulate adhesion, proliferation, migration and differentiation of cells) and to stimulate rapid bone formation [10,11,12]. The successful deposition of the conductive ink on the fibrous material depends on many factors, including capillary sorption and hydrophilic/hydrophobic properties of the printed surface [35]. In the case of the demonstrated study, it was possible to obtain on the porous surface the regular and sharp printed patterns, according to the assumed design. In vitro studies in body fluids have shown that the presence of conductive paths does not interfere with the formation of the apatite layer on the surface of the substrate during incubation in SBF, which was confirmed by SEM and FTIR studies. Therefore, based on the proposed composition, it is possible to obtain not only the material that is capable of mineralization, but also that has the possibility of electrical tissue growing stimulation. The features of scaffold play an important role in managing not only the cell adhesion, but also morphologies, and they can affect the intracellular responses [36]. There had been some studies demonstrating that the application of PANI as a conductive polymer did not affect the cellular behaviors [17,18,25]. These phenomena are also presented in our studies.

Microscopic observation of the surface of the materials produced during cell cultivation showed that cells grown on the test biomaterials take an elongated and spindle-shaped form and have longer and thinner extensions. No effect was observed of conductive paths made using PANI on changes in cell morphology; however, the testing so far has not included the cell cultivation upon the electrical stimulation.

The mineralization process in cell culture can be detected by the forming of mineralized nodules composed of inorganic hydroxyapatite. The OsteoimageTM assay, used for this research, contains a staining agent that binds to the hydroxyapatite part of the bone-like mineralized nodules deposited by cells and thereby can genuinely reflect the levels of cell mineralization. The progress of mineralization was better pronounced in the PCL/n-HAp sample, but after 21 days of cell culture on PCL/n-HAp/PANI scaffolds, a high mineralization capability for these materials was also confirmed. The amount of mineralization occurring in the PCL/n-HAp/PANI sample quantified on days 7 and 14 was similar to the control and much lower than for PCL/n-HAp. It may be that mineral deposition along the PANI paths is hindered since the scaffold (hydroxyapatite content) directly under them is less accessible to medium. This decreased accessibility to the medium may slow mineral precipitation and deposition at the paths relative to the non-modified part of the scaffold. However, osteoblast differentiation and mineralization after 21 days of cell culture increased with increasing culture time, following significantly higher calcium phosphate contents in the mineral deposition than the control group. One of advantages of conducting polymers used as scaffolds is electrical stimulation on the substrates. Scaffolds with conductive paths support cell proliferation and osteogenic differentiation. Based on the literature, we suppose that mineralization of the cells under electrical stimulation will dramatically increase compared to the materials without electrical stimulation.

In the future, it will be possible to use these materials as a tissue substrate which conducts electricity and thus stimulates in a planned manner the adhesion, proliferation, migration and differentiation of cells, and thereby accelerates the regeneration of bone tissue.

## 5. Conclusions

According to the presented results, the combination of the electrospinning process and inkjet printing is beneficial for producing nanofibrous and biomimetic substrates with conductive patterns on the surface. In vitro studies in physiological fluids have shown that the presence of conductive polymer deposited on the surface does not interfere with the formation of the apatite layer during incubation in SBF fluid. After 7 days of immersion in SBF, both materials were entirely covered by a dense apatite layer. Microscopic observations of the materials surfaces during cell culture showed that the cells cultured on both tested biomaterials have a similar elongated shape and were connected to each other via extensions, which allow for direct cell communication. The effect of PANI conducting pathways on changes in cell morphology was not observed. Cell differentiation was confirmed and cells showed a significantly improved mineralization progress within the contact with PCL/n-HAp/PANI materials. SEM images proved scaffolds’ biocompatibility. The materials described within this paper have potential to be used in the future as conductive tissue substrates and thus stimulating the adhesion, proliferation, migration and differentiation of cells in a planned manner, and thus accelerate the bone regeneration processes.

## Figures and Tables

**Table 1:** Assignment of FTIR bands for PANI [30].

Vibration Bond Range [cm^−1^]	Type of Vibration
600–900	Aromatic C-H out-of-plane bending vibration
1000–1180	Aromatic C-H in-plane bending vibration
1200–1300	C-N stretching of secondary aromatic ring
1400–1480	C=C stretching vibration of benzenoid (B) ring (N-B-N)
1500–1600	C=C stretching og quinoid (Q) ring (N=Q=N)

## Data Availability

Data is contained within the article.

## Supplementary Materials

The following are available online at https://www.mdpi.com/article/10.3390/ma14174837/s1.
