# Supplementary material for: Conductive Polyaniline Patterns on Electrospun Polycaprolactone/Hydroxyapatite Scaffolds for Bone Tissue Engineering
Source: Materials (Basel). 2021 Aug 26;14(17):4837. doi: 10.3390/ma14174837 (PMC8432661; doi:10.3390/ma14174837)
Supplement: Supplementary file 1 [file materials-14-04837-s001.zip › materials-1313087-supplementary.pdf]

Supplementary file.

Viability and proliferation of cells.

The viability and proliferation of cells (indirectly) on the test materials were examined using the CellTiter 96® AQueous One Solution Cell Proliferation Assay (Promega, USA) colorimetric assay. The active ingredients of the test are MTS tetrazole salt [3- (4,5-dimethyl-2-yl) -5- (3-carboxymethoxyphenyl) -2- (4-sulfophenyl) -2H-tetrazolium] and phenazine sulfate. The test is based on measuring the enzymatic activity of cells. NADH and NADPH dehydrogenases, active only in living cells, reduce the tetrazolium salt of MTS to colored formazan, the concentration of which is determined by colorimetry. The absorbance was read using a PolarStar reader. Results are presented in Figure A (below).

For PCL / n-HAp samples, the difference in cell viability between day 3 and day 7 is not statistically significant. However, the microscopic photos show fewer cells because the fibrous structure of the substrate makes it difficult for the cells to adhere and favors their penetration into the material.

In the case of samples with a PANI layer, a significant increase in cell viability was observed. The polyaniline layer and the associated flattening of the fibers and the compaction of the substrate structure (the patterns became more flattened and the fabric became more dense) probably promote the adhesion and flattening of cells on the fibrous substrate.

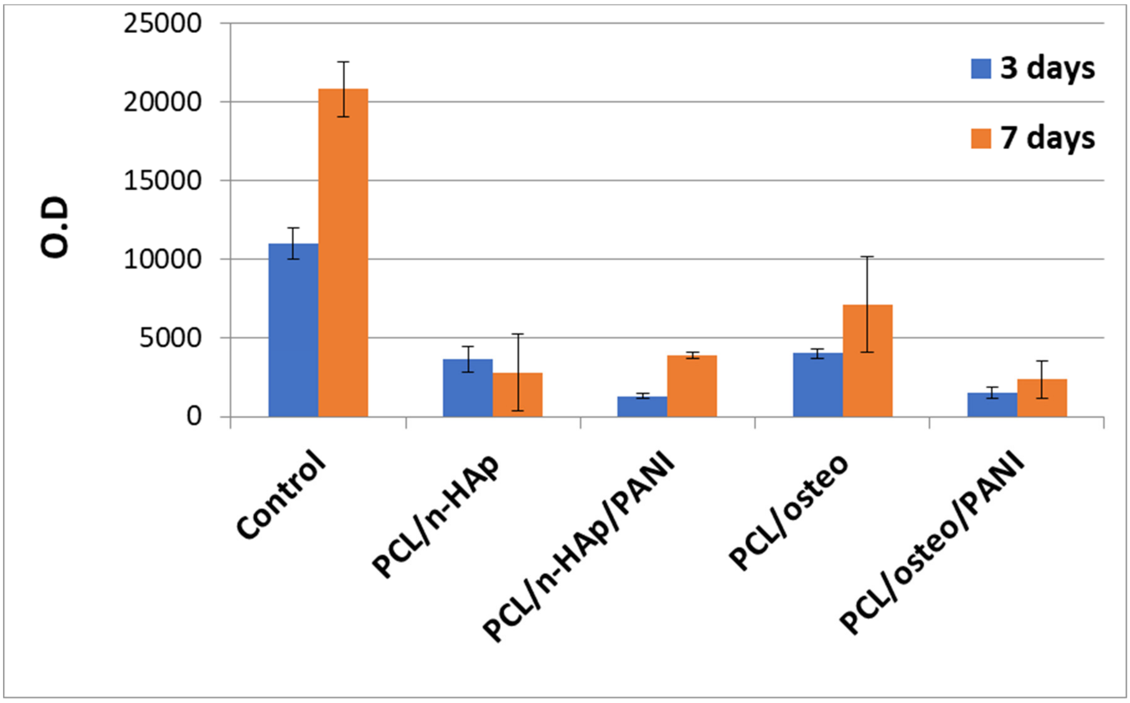

Figure S1. Results of the cell viability and proliferation.
